# A Biodegradable, Bio-Based Polymer for the Production of Tools for Aquaculture: Processing, Properties and Biodegradation in Sea Water

**DOI:** 10.3390/polym15040927

**Published:** 2023-02-13

**Authors:** F. Carfì Pavia, V. Brucato, M. C. Mistretta, L. Botta, F. P. La Mantia

**Affiliations:** 1 Dipartimento di Ingegneria, Università di Palermo, Viale Delle Scienze, 90128 Palermo, Italy; 2 INSTM, Consorzio Interuniversitario Nazionale di Scienza e Tecnologia dei Materiali, Via Giusti n 9, 50121 Firenze, Italy

**Keywords:** biodegradable polymers, marine water, biodegradation

## Abstract

Bio-based, biodegradable polymers can dramatically reduce the carbon dioxide released into the environment by substituting fossil-derived polymers in some applications. In this work, prototypes of trays for aquaculture applications were produced via injection molding by using a biodegradable polymer, Mater-Bi^®^. A characterization carried out via calorimetric, rheological and mechanical tests revealed that the polymer employed shows properties suitable for the production of tools to be used in aquaculture applications. Moreover, the samples were subjected to a biodegradation test in conditions that simulate the marine environment. The as-treated samples were characterized from gravimetrical, morphological and calorimetric point of views. The obtained data showed a relatively low biodegradation rate of the thick molded samples. This behavior is of crucial importance since it implies a long life in marine water for these manufacts before their disappearing.

## 1. Introduction

The United Nations Environment Programme (UNEP) defines “marine litter” as “any persistent, manufactured or processed solid material discarded, disposed of, or abandoned in the marine and coastal environment”, including items made of metals, wood, glass, polymeric materials and some other materials [1].

The presence of plastic debris in worldwide oceans is an important concern for the environment [2]. The key sources of microplastics in marine systems are cosmetic and cleaning products discharged in domestic wastewaters. Nonetheless, these are not the sole sources of microplastics in the environments. Additional sources include those of industrial origin, such as feedstocks used in the manufacturing of plastic products and from spillage of plastic resin powders or pellets used for airblasting [3]. Indeed, due to their remarkable resistance to degradation in sea water [1,4,5,6,7,8], their life can be very long and cause damages to the marine life. Moreover, beyond plastic objects, many tools for fishing or for aquaculture, such as ropes, net, cages, etc., are often left in the sea, strongly increasing the amount of plastic debris in the oceans. Just like plastic tools, other tools made of other materials such as metals are left in the ocean, increasing the amount of marine litter and the environmental impact. The use of plastic materials can effectively improve the handling of the cages, reducing the weight and cost of the installation.

To limit marine plastic pollution, many solutions have been proposed on prevention, collection and identification, with scarce importance on the development of treatment/recycling options [9].

The National Project “PON PLACE” [10] aims to test cutting-edge technologies and solutions for the eco-sustainable reuse of offshore platforms at the end of their productive phase. One possible use of the platform is the design, development and demonstration of the effectiveness of innovative eco-sustainable strategies of multi-trophic aquacultures based on integrated shellfish and holothurian. In this way, an important item is the development and testing of prototype biopolymers artefacts for marine aquaculture for the replacement of materials currently used to produce ropes, nets, trays and cages for marine cultivations with renewable materials. In this way, our attention has been focused, in particular, on the production of cages and trays for the aquaculture of oysters and sea cucumber made at present by metals.

As a matter of fact, the use of biodegradable polymers can avoid any environmental impact due to the fragmentation of the manufacts because of the photo-oxidative degradation with well-known consequences on the marine environment.

Biodegradable polymers can play an important role in the strategy of decreasing the environmental impact of plastic objects in the oceans. Indeed, the use of biodegradable polymers can significantly improve the managing of the end-of-life of these tools to produce anthropogenic CO_2_ in the environment with a consequent increase of the green-house effect. The use of bio-based, biodegradable polymers, on the contrary, not only resolve the end-of-life of the polymers, but also importantly contribute to the decarbonization of plastics.

The main mechanisms leading to polymer degradation in the marine environment, and therefore to the formation of secondary microplastics, are photo-oxidation, thermo-oxidative degradation, hydrolytic degradation, biodegradation and mechanical degradation [11]

Indeed, biodegradable polymers, after their lifetime, are degraded by microorganisms yielding CO_2_, H_2_O and biomass and without any negative environmental impact. Finally, bio-based, biodegradable polymers coming from renewable biomasses also avoid the use of fossil-derived polymers, improving the decarbonization of plastics.

There are several bio-based and biodegradable polymers available on the market, such as starch-based bioplastics, polylactic acid (PLA), polyhydroxy butyrate (PHB) and polyhydroxyalkanoates (PHA) [12]. Another class of contemporary biodegradable polymers are surely biocomposites, e.g., the Poly(3-hydroxybutyrate-*co*-3-hydroxyvalerate) (PHBV)/natural fibers [13] or Mater-Bi/Biochar [14]-based biocomposites.

Mater-Bi is one of the most commercialized bio-based, biodegradable families of polymers used in many applications. Several interesting studies concerning the degradation of Mater-Bi (pellets or thin films) have demonstrated both its faster biodegradability with respect to traditional plastics, and a slower biodegradation rate in marine environmental conditions other than in soil [15,16,17,18]. Moreover, these studies suggest that Mater-Bi does not generate persistent microplastics, because, as erosion increases the surface area, this in turn increases the biodegradation rate to levels similar to those required by the OECD for chemicals to be defined as readily biodegradable. Finally, it has been shown that Mater Bi seems to not induce adverse effects on representative aquatic organisms [19].

The aim of this work is to investigate the possible use of an injection molding grade Mater-Bi sample for the production of these trays.

The sample of biodegradable polymer chosen for this study has been successfully molded, showing appropriate rheological and mechanical properties for this application. A molding grade polypropylene sample used for the production of trays has been also investigated for comparison.

The reason for the choice of this selected grade of Mater-Bi is that it is a bio-based, biodegradable polymer system and one of the most commercialized starch-based blends used in biodegradable applications. An understanding of the degradation mechanisms of plastics in the environment, and in particular in the marine environment, is complicated by the complexity and extent of the environmental matrix and the boundary conditions [1]. For that reason, a preliminary biodegradation test in simulated sea water was also carried out. The sample of Mater-Bi has been easily injection-molded and shows good mechanical properties. The biodegradable polymer thick injection-molded samples degrade slowly in the simulated marine environment, and this is a positive point because the life in the sea water of these bio-based, biodegradable trays is very long and could decrease the cost of the installation.

## 2. Materials and Methods

### 2.1. Materials

The polymers used in this work are reported in Table 1, as is the Melt Flow Index (MFI), as reported in the technical datasheets.

PP is a polypropylene copolymer suitable to injection molding (IM) operations and used as a reference sample (for comparison purposes). Mater-Bi has proprietary composition, but, as reported in the literature, is a biodegradable starch-based polymer [20].

Due to possible hydrolytic chain scission during processing, Mater-Bi was dried for 4 h under vacuum at 60 °C.

### 2.2. Preparation of the Samples

The two polymers were subjected to injection molding in an industrial injection molding machine, Engel 260. The main processing parameters are shown in Table 2.

The specimens for the tensile tests on the raw polymers were manufactured by compression molding (CM) in a laboratory hydraulic press (Carver, Wabash, IN, USA). The compression molding temperature was set at 190 °C for the PP and at 180 °C for MB. Compression time was approx. 4 min, under a load of about ≈5 MPa. Before compression molding, the Mater-Bi pellets were dried in a vacuum oven for 4 h at 60 °C.

### 2.3. Characterization

The thermograms of the raw polymers and of the injection-molded polymers were recorded with a Seteram DSC131 Evo. The heating rate was 10 °C/min in a temperature range of 25 to 200 °C. To take into account the morphology acquired during processing, the melting enthalpy was measured during the first heating step.

Rheological characterization in shear flow was performed by using a capillary rheometer Rheologic 1000 (CEAST, Torino, Italy), equipped with the following capillary geometry: a diameter equal to 1 mm and a length-to-diameter (L/D) of the capillary ratio equal to 40. Due to high L/D ratio, the Bagley’s correction was neglected, whereas the Rabinowitch’s correction has been applied throughout. The test temperature was 235 °C for both the polymers, the same temperatures adopted in the injection molding machine. Before rheological testing, the Mater-Bi pellets were dried in a vacuum oven for 4 h at 60 °C.

**Figure 1:**
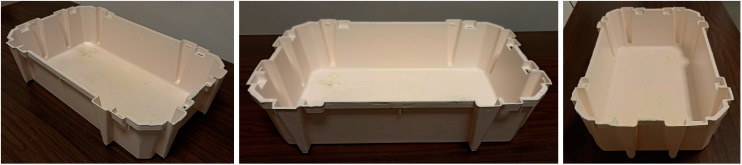
Injection-molded tray. All the specimens utilized for mechanical and biodegradation tests were obtained from the flat areas of the sides of the tray.

Mechanical characterization of all the samples was performed according to ASTM D638-14 in tensile mode using the universal testing machine Instron mod. 3365 (Instron, High Wycombe, UK) with a load cell of 5 kN, at a crosshead speed of 1 mm/min until a deformation of 3%, and then at a crosshead speed of to 100 mm/min until final rupture. The reproducibility of the results was good (±6%). Tensile characterization was performed both on compression-molded (CM) samples and on the specimens directly cut out from the side face of the injection-molded (IM) tray (Figure 1).

Impact tests were carried out on the injection-molded samples in unnotched Izod mode by using a Instron (Norwood, MA, USA) Ceast 9050 universal pendulum at 15 J (with a ±8% reproducibility). The dimensions of the specimens were: length: 12 cm; width: 1.5 cm and thickness: 0.3 cm. The reported mechanical values are an average of at least seven tests.

### 2.4. Biodegration

The samples were cut from the side faces of the molded trays. Square samples of about 2 cm were obtained. The samples were kept in an oven at 40 °C and under vacuum overnight, to prevent any water absorption, and weighed with a SECURA224-1S analytical balance (Sartorius). Then, each sample was put into a petri dish filled with artificial sea water (ASW) and prepared according to ASTM D1141-52. Distilled water (DW) was used as positive control since it has been demonstrated that the degradation rate of polymers changes into the two media. Specifically, the degradation is faster in distilled water than in seawater [21]. The measured pHs for the two media were 8.2 for ASW and 6.7 for DW.

The petri dishes were maintained in an incubator at a temperature of 15 °C for the whole experiment.

At regular intervals (2, 4, 7, 12 and 18 weeks), at least seven samples for both conditions were extracted, abundantly rinsed in deionized water to totally remove the salt from their surfaces and finally kept overnight in an oven at 40 °C in order to remove any trace of water. The as-treated samples were then weighed again.

The percentage decrease in weight (*W_loss%_*) of the samples was calculated according to the following relationship:

(1) $$W_{loss\%}=\frac{W_{i}-W_{f}}{W_{i}}\cdot 100$$

where *W_i_* and *W_f_* are the initial weight and the weight after the immersion of each sample, respectively.

SEM images were recorded with a QUANTA 200F, FEI (Thermo Fisher, Waltham, MA, USA) scanning electronic microscope with an accelerating voltage of 10 kV. Before imaging, the samples were gold-sputtered (Sputtering Scancoat Six, Edwards) for 120 s under an argon atmosphere. SEM images were exported as 24-bit image files using the tagged image file format (tiff).

A calorimetric analysis was carried out in a DSC Setaram 131 Evo (Setaram Inc., CH). The treated samples were reduced in size (2 mm × 2 mm), weighed, inserted into an aluminium pan and analysed under a nitrogen gas flow of 1 mL/min. The following thermal history was set up for every sample:

- Stabilization at 10 °C for 600 s;
- heating at 10 °C/s from 10 to 210 °C;
- maintaining at 210 °C for 600 s;
- cooling at 10 °C/s from 210 to 30 °C.

The melting and crystallization temperatures and enthalpies were obtained analysing the obtained thermograms with “Calisto data processing” software, provided by the instrument manufacturer.

### 2.5. Statistical Analysis

Statistical analyses of the data were performed through one-way analysis of variance and, when applicable, data were compared using the Student’s *t*-test. *p*-value < 0.05 was considered statistically significant.

## 3. Results and Discussion

### 3.1. Characterization of the Biodegradable Polymer and of the Trays

**Figure 2:**
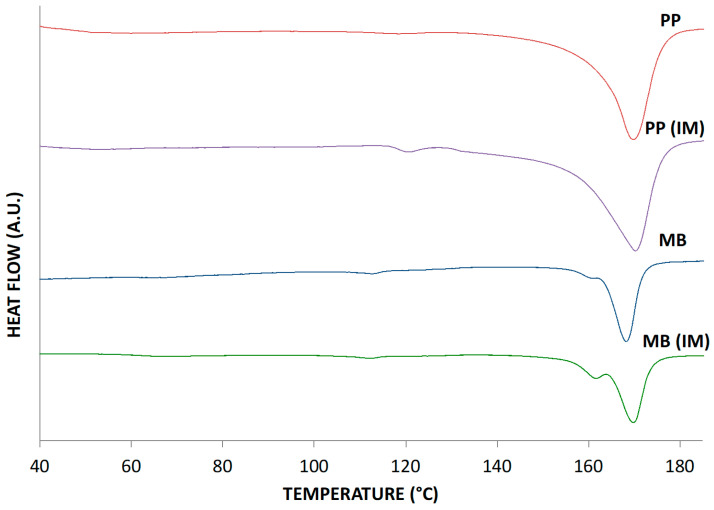
Thermograms of the raw Mater-Bi and polypropylene and of the same molded polymers.

In Figure 2, the thermograms of the raw samples and of a fragment of the IM samples are reported.

Both polymers show a semicrystalline morphology and both polymer shows a slight but detectable increase of the crystallinity and of the melting points after injection molding; see Table 3.

It is then evident that the injection molding operation improves the order of the macromolecules of the polymers, giving rise to a better crystallinity morphology, as evidenced by the rise of the melting temperature as well.

**Figure 3:**
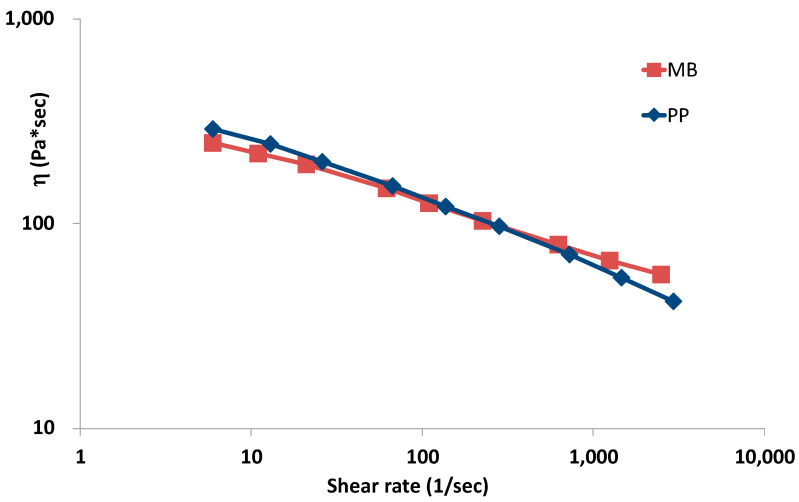
Rheological curves of the two polymers.

The flow curves obtained by means of the capillary rheometer are shown in Figure 3. The flow curves have been measured in the range of shear rates because it is the typical shear rate range encountered in industrial operations of injection molding. At this temperature, the flow curves of the two polymers are almost superimposable. However, at the lowest shear rates (about 10 s^−1^), the biodegradable polymer shows a slightly lower viscosity, while, at the very high shear rates, typical of the injection moulding operation, the viscosity of the biodegradable polymer is slightly higher than that of the polyethylene. Of course, these curves, even if measured at lower shear rates, cannot provide any information about the different molecular structure of the two polymers because the biodegradable polymer is a blend. These flow curves put in evidence that when the two polymers are processed in an injection molding operation at the injection temperature of about 235 °C, the holding pressure and the other injection processing parameters should be about the same (see Table 3).

### 3.2. Mechanical Characterization

The main tensile properties (elastic modulus, E, tensile strength, TS, elongation at break, EB, of compression-molded samples are compared in Table 4, where E, TS and EB are reported. In the same Table, the values of the injection-molded samples (IM) are also reported.

The specimens of the injection-molded samples were cut from the bottom of the tray (see Figure 1).

The biodegradable sample is more rigid than the PP sample because it shows higher values of elastic modulus and tensile strength and lower values of the elongation at break for both compression- and injection-molded specimens. The elastic modulus and tensile strength of the injection-molded specimens are slightly higher than those of the compression- molded specimens, while the elongation at break is slightly lower. This is due to the different morphology of the two types of samples. Indeed, the increased rigidity of the injection-molded samples can be ascribed to the increase of the crystallinity as reported before.

In addition to tensile tests, impact tests were performed on the injection-molded systems, as seen in Table 5. The tests have been performed on samples obtained from the trays, as with the ones for the tensile tests. Although the dimensions of these samples are slightly different from those of the standardised tests, the tests have been carried out just to compare the values obtained for the two polymers. The value relative to the biodegradable polymer is lower than that of the copolymer, but certainly appropriate for the proposed use.

### 3.3. Biodegradation Tests

**Figure 4:**
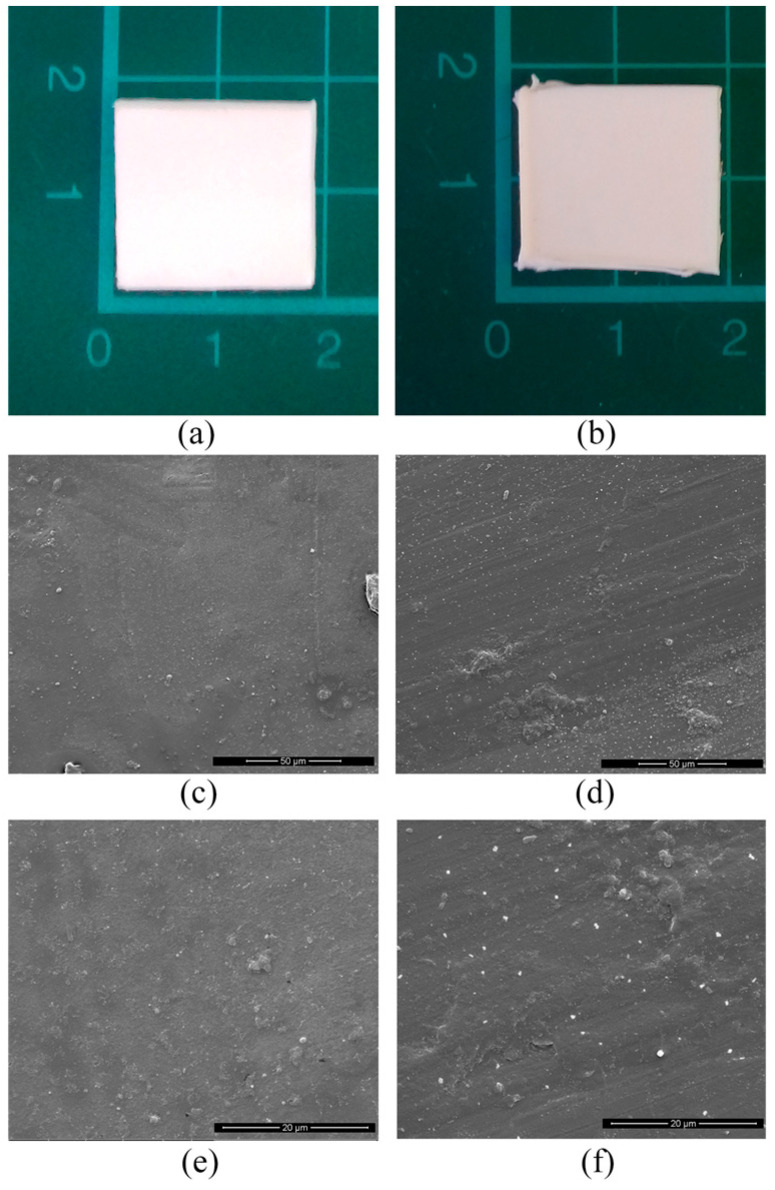
Sample before (**left**) and after (**right**) incubation in artificial sea water. Macroscopical view (**a,b**); Scanning Electronic Microscope (SEM) micrographs (**c**–**f**)**. SEM** magnifications: (**c**,**d**) 1000×; (**e**,**f**) 5000×.

Figure 4a,b shows the samples before and after the degradation test. From the macroscopical point of view, the two samples appeared identical. In order to detect differences from a microscopic point of view, the same samples were observed through Scanning Electronic Microscopy. SEM micrographs of the surface of the samples have revealed a totally flat surface in the untreated sample (Figure 4c,e). An almost same morphology was also observed at 18 weeks in the ASW-treated sample at lower and higher magnifications (Figure 4d,f). The same morphology was detected on DW-treated samples (data not shown). However, a detectable difference in surface morphology is the presence of an increased roughness, which indicates the beginning of degradation of the materials. A very similar structure was observed in a recent paper in which an extensive study of PLA surface degradation in aqueous media was carried out [22].

**Figure 5:**
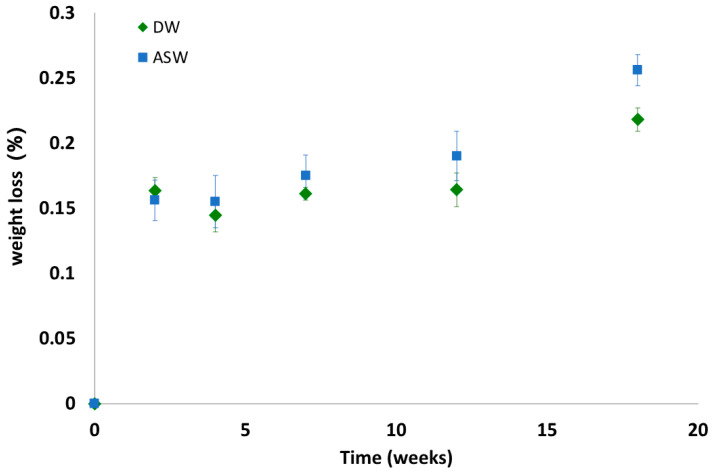
Weight loss of the incubated samples in the two degradation media (DW: distilled water; ASW: artificial sea water).

Figure 5 shows the weight loss data obtained after 18 weeks of incubation in DW and ASW. In both cases, the weight differences remained below 0.2%. The differences in weight loss recorded between the samples incubated in distilled water and artificial seawater are not statistically significant for all time-points.

**Figure 6:**
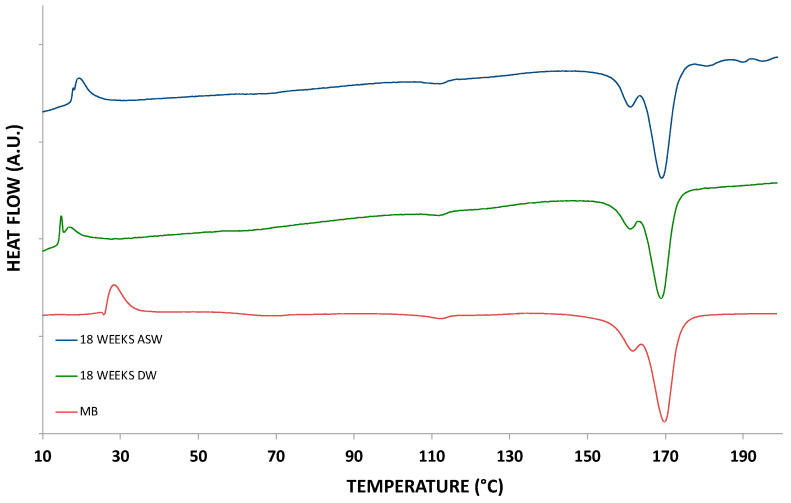
DSC heating thermograms of the incubated and non-incubated samples (DW: distilled water; ASW: artificial sea water).

**Figure 7:**
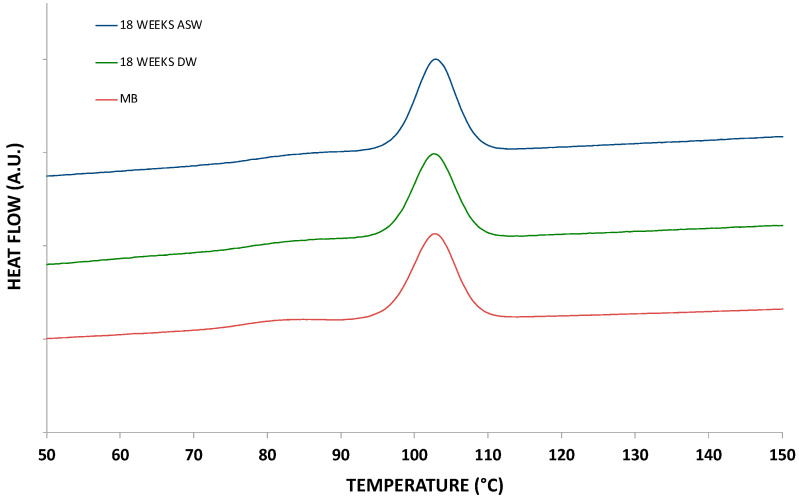
DSC cooling thermograms of the incubated and non-incubated samples (DW: distilled water; ASW: artificial sea water).

The graphs in Figure 6 and Figure 7 show the thermograms relating to heating and cooling, respectively, of the not-incubated (molded samples used as control, maintained in air in an environment similar to that of the soaked samples) and incubated samples. Regarding heating, there are no significant differences in the calorimetric profiles, except for a more defined melting peak in the immersed samples. The same behaviour was detected for the thermograms of the cooling ramp. Furthermore, in this case, the data confirm the absence of differences between the immersed and non-immersed samples from a calorimetric point of view.

From the thermograms it was possible to derive for each sample both the melting enthalpy and temperature, as well as the crystallization enthalpy and temperature.

These values, reported in Table 6, indicate a substantial equivalence between the non-immersed and immersed samples, indicating that the permanence in the degradation media did not significantly affect the calorimetric properties of the materials. In addition, as in the weight loss analyses, slight differences were registered between the samples degraded in DW and in ASW.

All things considered, all the analyses carried out have highlighted a modest level of biodegradation of the samples after 18 weeks of incubation.

A study, conducted by Niaounakis et al., focused on the degradation of Mater Bi films in marine environments, has shown that after 12 months, a Mater Bi film undergoes severe deterioration due to a water uptake followed by the loss of plasticizer and progressive hydrolysis of the starch component of the material [23]. Another interesting attempt to study the marine degradation of Mater Bi was carried out by O’Brine and Thompson. In this work, a massive fragmentation of Mater-Bi and its dispersion in free seawater after 40 weeks was noticed, whereas other materials tested lost only about the 2% of their surface area [24].

In our case, a slight weight loss coupled with a substantial absence of change in crystallinity of the samples were observed. Conversely, a detectable increase of roughness of the surface leads to propose a surface degradation mechanism that does not depend, at least for the time taken in consideration, by the degradation media. As a matter of fact, a faster degradation in distilled water other than in seawater, due to lower content of oxygen in ASW, should be expected [25].

The relatively low biodegradation rate of the polymer detected in this study can be related to the thickness and to the shape of the tested samples. It was recently shown as the thickness is a fundamental variable in the degradation tests [26].

Other factors could be the low content of oxygen in the water, in particular in sea water, and the resulting absence of photo-oxidation (that leads to the weakening and fracturing of polymers) [27]. Finally, it is important to underline the absence of microorganisms that, as is well known, rapidly colonize the plastics introduced in seawater through their aerobic processes [28]. Hence, the experimental setup used in this study does not reproduce many of the effects that function in the open sea: waves, currents, solar irradiation in the case of superficial floating, etc. Therefore, field testing should complement the approach proposed in this study [18].

However, to our knowledge, this preliminary test is the first example of a study on the degradation of a Mater-Bi-molded product in conditions that simulate a marine environment.

## 4. Conclusions

Tools for fishing and aquaculture are mainly made of fossil-derived, unbiodegradable polymers (ropes, nets, etc.) or metals (cages, trays, etc.). Those materials can give rise to many environmental problems. Plastics are degraded due to photo-oxidation, and the consequent fragmentation disperses dangerous debris in the sea. The use of bio-based, biodegradable plastics instead of both unbiodegradable plastics can remove all of these problems, since their pollutants degrade very quickly. The biodegradable plastic is biodegraded yielding CO_2_, water and biomass useful to the aquatic life. In this work, we have demonstrated that a bio-based, biodegradable polymer can be processed for obtaining trays for aquaculture. The obtained molded manufacts showed acceptable mechanical properties and present a biodegradation rate in water that is relatively low due to a surface degradation mechanism. This behavior can be considered very useful since the duration of these trays in marine water is long, decreasing the environmental impact and the cost of the installation.

## Figures and Tables

**Table 1:** Materials used in this work and related codes.

Material	Manufacturer	MFI, g/10 min	Sample Code
Yuplene BH3821	SK Global Chemical (Korea)	28	PP
Mater-Bi EI51N0	Novamont (Italy)	35	MB

**Table 2:** Main injection molding process parameters.

Sample	Temperature Profile, °C	Injection Pressure, Bar	Holding Pressure, Bar	Mold Temperature, °C	Flow Rate, cm^3^/s	Holding Time, s
PP	170/185/215/235	80	35	30	400	60
MB	170/180/205/235	90	40	30	330	60

**Table 3:** Enthalpy of fusion and melting temperatures of the raw polymers and of the injection- molded polymers.

Sample	Enthalpy of Fusion, J/g	Melting Temperature, °C
PP	64.1	169.6
PP (IM)	75.5	170.1
MB	27.1	168.2
MB (IM)	29.5	169.8

**Table 4:** Elastic modulus, E, Tensile strength, TS, and Elongation at break, EB, of the compression- molded (CM) and injection-molded (IM) samples.

Sample	E, MPa	TS, MPa	EB, %
PP	1040 ± 66	20.6 ± 1.2	6.9 ± 0.7
PP (IM)	1154 ± 61	21.8 ± 1.5	5.8 ± 0.5
MB	1910 ± 142	36.60 ± 2.2	3.4 ± 0.5
MB (IM)	2120 ± 131	38.20 ± 2.8	2.8 ± 0.3

**Table 5:** Impact strength of the injection-molded (IM) samples.

Sample	IS, J
PP (IM)	7.45
MB (IM)	4.84

**Table 6:** Melting and crystallization enthalpies and temperatures of the incubated samples at 18 weeks.

Sample	Melting Enthalpy (J/g)	Melting Temperature (°C)	Crystallization Enthalpy (J/g)	Crystallization Temperature (°C)
MB (IM)	29.5	169.8	27.7	102.7
18 WEEKS DW	29.5	168.9	27.7	102.8
18 WEEKS ASW	29.7	169.1	28.0	102.1

## Data Availability

Not applicable.
